# Human Neurobehavioral Effects of Long-Term Exposure to Styrene: A Meta-Analysis

**DOI:** 10.1289/ehp.7518

**Published:** 2005-01-27

**Authors:** Vernon A. Benignus, Andrew M. Geller, William K Boyes, Philip J. Bushnell

**Affiliations:** ^1^Human Studies Division, National Health and Environmental Effects Research Laboratory, Office of Research and Development, U.S. Environmental Protection Agency, Research Triangle Park, North Carolina, USA;; ^2^Department of Psychology, University of North Carolina at Chapel Hill, Chapel Hill, North Carolina, USA;; ^3^Neurotoxicology Division, National Health and Environmental Effects Research Laboratory, Office of Research and Development, U.S. Environmental Protection Agency, Research Triangle Park, North Carolina, USA

**Keywords:** choice reaction time, chronic, color perception, long-term, meta-analyses, neurobehavioral, review, styrene, workplace

## Abstract

Many reports in the literature suggest that long-term exposure to styrene may exert a variety of effects on the nervous system, including increased choice reaction time and decreased performance of color discrimination and color arrangement tasks. Sufficient information exists to perform a meta-analysis of these observations quantifying the relationships between exposure (estimated from biomarkers) and effects on two measures of central nervous system function: reaction time and color vision. To perform the meta-analysis, we pooled data into a single database for each end point. End-point data were transformed to a common metric of effect magnitude (percentage of baseline). We estimated styrene concentration from biomarkers of exposure and fitted linear least-squares equations to the pooled data to produce dose–effect relationships. Statistically significant relationships were demonstrated between cumulative styrene exposure and increased choice reaction time as well as increased color confusion index. Eight work-years of exposure to 20 ppm styrene was estimated to produce a 6.5% increase in choice reaction time, which has been shown to significantly increase the probability of automobile accidents. The same exposure history was predicted to increase the color confusion index as much as 1.7 additional years of age in men.

Chronic exposure to styrene and a number of other volatile organic compounds has been linked to the occurrence of neurologic and behavioral deficits, including increased reaction time (Kishi et al. 2000), visual system disturbances (Iregren et al. 2002), and neurophysiologic alterations (e.g., Harkonen et al. 1978; Lilis et al. 1978; Seppalainen and Harkonen 1976; Stetkarova et al. 1993). In addition, a number of dosimetric studies have quantified the relationship between inhaled styrene and markers of exposure: urinary concentrations of styrene (Ghittori et al. 1987; Gobba et al. 1993; Imbriani et al. 1986, 1990; Pezzagno et al. 1985) or mandelic acid, its major excretory metabolite (Campagna et al. 1995; De Rosa et al. 1993; Fields and Horstman 1979; Severi et al. 1994). The findings in this literature suggest that styrene may exert a variety of effects on the nervous system and that sufficient dosimetric information exists to develop quantitative relationships between these effects and conditions of exposure. The purpose of this study was to perform a meta-analysis of these observations, to quantify the relationship between exposure (estimated from the biomarkers) and effects on two measures of central nervous system function: reaction time and color vision. An effort was made to assess the importance of styrene-related deficits to real-world task performance. An insufficient number of reports were found for meta-analyses of other neurotoxic effects.

## Behavioral Outcomes

Reaction time has been a popular variable for assessment of impaired behavioral task performance for several reasons, including ease of measurement, adversity of effect, and sensitivity to drugs and toxic chemicals. Reaction time tasks are usually divided into simple reaction time (SRT), in which the subject must simply react to a predefined stimulus as quickly as possible, and choice reaction time (CRT), in which the subject must first select between options before deciding whether or in what way to respond.

Long-term exposure to a number of solvents has been reported to also produce deficits in the performance of screening tests for perception of color or visual contrast (Geller and Hudnell 1997; Iregren et al. 2002). Perhaps the most prevalent agent in this growing literature is styrene, for which numerous published studies have reported that exposure was associated with visual deficits, in particular, an acquired impairment of color perception. Color vision was usually assessed using the Lanthony desaturated D-15d color vision test (Lanthony 1978), a hue discrimination test designed to grade the loss of color discrimination from mild to moderate. Performance on the test was usually quantified using the color confusion index (CCI) scale (Geller 2001). Details of testing procedure and scoring are reviewed in Geller and Hudnell (1997).

## Meta-Analyses

The literature reporting the effects of long-term styrene exposure on behavioral performance is diverse. Critical experimental factors such as group sizes, analytical approaches, and methodologic details vary greatly across studies. A meta-analysis of the literature involving a quantitative treatment of the combined data from a number of individual reports can help unify the literature by improving *a*) accuracy, by minimizing the impact of single, perhaps anomalous reports; *b*) precision, by including a large number of subjects; and *c*) generality, by aggregating a variety of studies with differing subject populations and exposure histories.

A meta-analysis of the effects of exposure to styrene and other organic solvents on CCI was recently reported by Paramei et al. (2004). This analysis featured means from various studies that were converted to *Z*-scores to place them on a common scale of measurement for comparison and to help assess the possibility that an effect might have been statistically significant. High variance was observed between the results of different studies after transformation, and the authors argued that no reliable conclusions could be drawn about the effects of styrene on CCI. The *Z*-score transform, however, compares the magnitude of each particular effect (the mean) with the variance for that measure. This transform conflates the measure of magnitude of an effect with its stability, so a transformed score may be larger or smaller depending on the variance of the group.

In the present work, our intent was to evaluate the magnitude of styrene effects on CCI and reaction times. Thus, values were not converted to *Z*-scores but were expressed as a percentage of baseline. The dose levels were expressed as inhaled-air styrene concentration multiplied by the work-years of exposure. These data were then fitted to a dose–effect regression equation. In this analysis, magnitude estimates were not conflated with variances, and the variation among the reported data was used to compute confidence limits and test the fitted result for statistical significance (Benignus et al. 1998).

## Materials and Methods

### Estimates of Exposure

In the neurobehavioral reports, four different measures were used to quantify the level of exposure to styrene: *a*) concentration of styrene in inhaled air (personal monitors) given as a time-weighted average (TWA), *b*) concentration of styrene in urine, *c*) concentration of mandelic acid in urine as a fraction of creatinine in urine, and *d*) concentration of mandelic acid in urine. Individual-subject data were given via scatter plots in some studies, whereas only group means were provided in others. In the interest of homogeneity, we computed group means from individual-subject data and used them to express inhaled-air styrene concentration. We also analyzed individual-subject exposure concentrations as a check on the results obtained from means.

To pool the data on effects of exposure across studies, it was necessary to convert data from the reported measurement of exposure to one common scale (inhaled-air styrene concentration). To estimate inhaled-air styrene concentration from the measures supplied by the original authors, we found representative publications in which the various methods of reporting urinalyses were standardized and tested (Table 1). In these publications, styrene measurements from personal monitors were recorded for a work shift, and then urine was sampled after exposure. We digitized plotted data for individual subjects and pooled them into common databases, one for each of the three methods of urine analysis. We then fitted regression equations to the pooled data to predict inhaled-air styrene concentration from the various observed urine measures.

In neurobehavioral studies, exposure to styrene was usually estimated at the end of a work shift. It was implicitly assumed by the authors of the reviewed articles that this single measure was a representative measure of the workers’ exposure history. This measure of exposure would underestimate historical exposure in cases where there have been improvements in environmental controls in the workplace (Gong et al. 2002).

Duration of exposure in work-years was given in all of the neurobehavioral reports, but always as group means, even when individual-subject data were given for styrene concentration. The mean duration of exposure and mean concentration, although fixed for a particular report, varied across reports. Thus, when pooling data from several reports, we were able to analyze for the effects of a combination of concentration and duration of exposure, expressed as the product of concentration and time (ppm work-years) (Cavalleri et al. 2000; Haber 1924; Miller et al. 2000).

### Measures of Adverse Outcomes

In each neurobehavioral study, tests were given at a fixed time after the last exposure, usually the morning after the work shift of the previous day; occasionally a weekend elapsed between the last exposure and behavioral testing. This delay was intended to avoid possible acute effects of the parent compound or its metabolites.

Most of the reports did not specify whether the personnel administering the tests were aware of the group to which each subject belonged (Tables 2, 3). Such “nonblind” procedures have the potential to overestimate the effects of a toxicant in laboratory experiments (Benignus 1993).

Four publications produced a total of seven data points for CRT (Table 2). For SRT, three articles yielded six data points (Table 3). Some studies provided data on both SRT and CRT. Only those studies of color vision that employed the Lanthony desaturated D-15d test were used (Table 4). The data were individual CCI and styrene exposure estimates from five studies. The illuminants used in testing were of intensity 1,000–1,200 lux with spectral distribution specified as “daylight illumination.” Color vision testing was done in the morning, before the work shift in four of five of the references used. CCI was reported as a raw score in three of the five studies; in the other two, CCI was adjusted for individual age and alcohol consumption. In the three studies reporting raw CCI scores, subjects were eliminated from analyses if criteria were exceeded for age, disease, alcohol consumption, or drug use.

### Quantitative Procedures

When data were graphically presented in publications, graphs were digitized as previously described (Benignus et al. 1998). Graphs were scanned and imported into digitization software (UN-SCAN-IT; Silk Scientific, Orem, UT), which produced a table of *x,y*-coordinates for each data point. Because some of the points on a plot were hidden behind others, the number of digitized data points was always slightly less than the number of points that the original authors reported. The number of digitized points was used and reported in the present work. When data were given numerically by the authors, these were used directly.

#### Normalization of data before pooling.

Data from different studies can be pooled only if the measurement scales can be made comparable. Each datum was adjusted as follows:


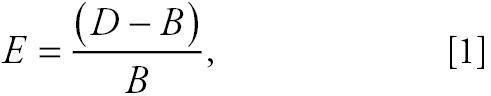


in which *E* is a normalized (effect) value, *D* is the value of the unadjusted dependent variable, and *B* is the value of a baseline condition. In studies where only means were reported, the performance of the control group provided baseline values. In some other studies reporting individual-subject data, independent control groups were not studied, and the investigators relied on a dose–effect analysis in which subjects with very low exposures served as implicit controls. In the present work, when individual-subject data were given, the mean of all data from exposed to < 10 ppm styrene was used as a baseline. This procedure was followed even if specific control groups were measured to assure consistency and also to include the maximum number of studies in the meta-analysis.

#### Fitting dose–effect curves.

The data were pooled after all useable data had been transformed by Equation 1 and all exposure data had been converted to inhaled-air styrene. A linear regression equation of the form


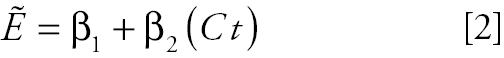


was then fitted to the data. Here *Ē* is the estimated value of the effect, *C* is the concentration of styrene in inhaled air (parts per million), *t* is the duration of exposure (work-years), and the βs are empirical parameters fitted with a least-squares procedure (Proc REG; SAS Institute Inc., Cary, NC). Equation 2 was fitted first to assure that the intercept term, β_1_, was near zero and not statistically significant (which should be the case for data adjusted by Equation 1). If the intercept term was not statistically significant, Equation 2 was refitted with only a slope (β_2_) term.

In cases where the regression lines were fitted to means from various studies or groups, each mean was weighted by the number of subjects for that mean. This was done by the “weight” statement of Proc REG. This had the effect of giving the larger studies (with smaller SEs) greater weight in the fitting procedure. If a regression equation was found to be statistically significant, the data were plotted with the effect on the y-axis and with the product of styrene concentration (in parts per million) and work-years on the x-axis. In general, when regression lines are fitted to means instead of individual-subject data, estimated lines are very nearly the same, but confidence limits will be somewhat wider than if individual-subject data had been available. This may be intuitively explained as due to the loss of information when means are used. The effects were also plotted separately as functions of styrene parts per million alone with four lines for 2, 4, 6, and 8 work-years of exposure. These lines were calculated by solving the regression equation with styrene concentration as the independent variable for each of the work-years of exposure (either 2, 4, 6, or 8). Because some of the published reports gave SRT and CRT data only as means ± SDs, all regression lines were fitted to means, even for CCI, where individual-subject data were available. For the CCI data, means were computed from the baseline-adjusted individual-subject data by dividing the exposure range of each report into two or three subranges and computing the means of exposure and effect magnitude within the subranges. To assess the effect of converting individual-subject data to means, a regression line was also fitted to the individual-subject data.

## Results

### Estimating Exposure

**Figure 1:**
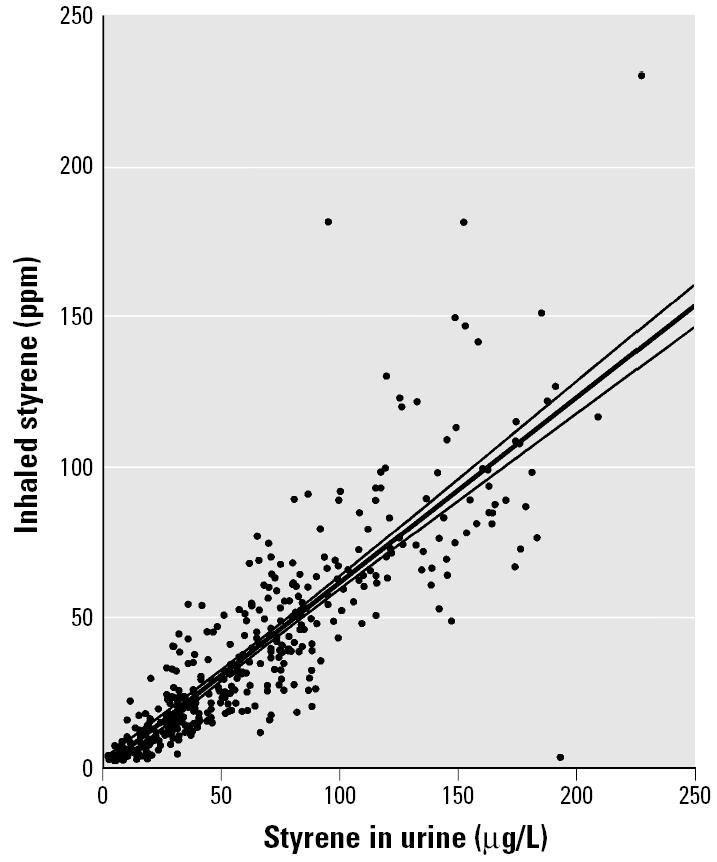
Scatter plot and fitted regression line with 95% CLs for estimation of styrene concentration in inhaled air (TWA ppm) from styrene concentration in urine. Digitized individual-subject data were pooled from five studies.

**Figure 2:**
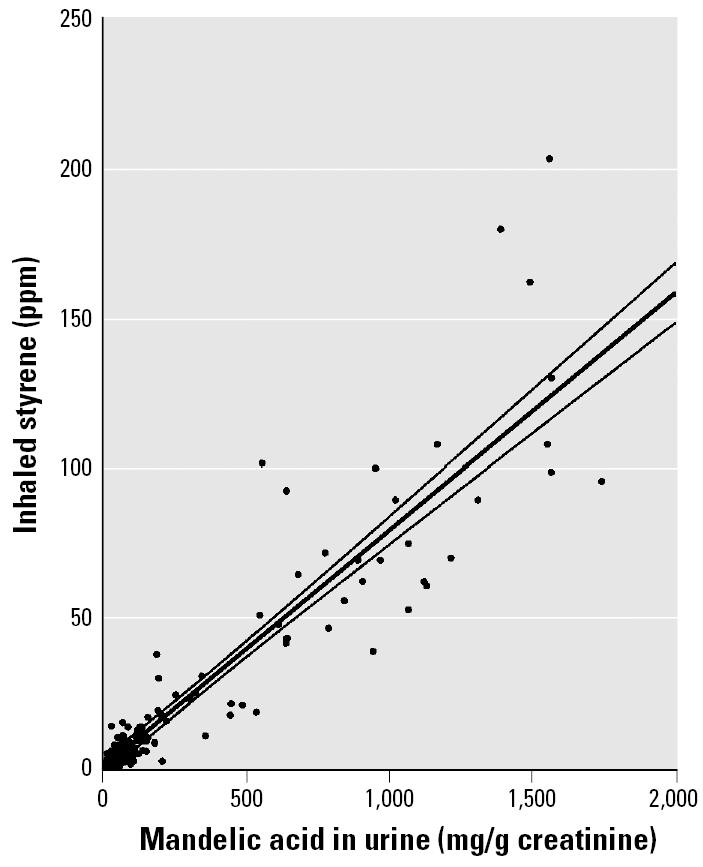
Scatter plot and fitted regression line with 95% CLs for estimation of styrene concentration in inhaled air (TWA ppm) from mandelic acid in urine, normalized by creatinine (mg/g). Digitized individual-subject data were pooled from three studies.

**Figure 3:**
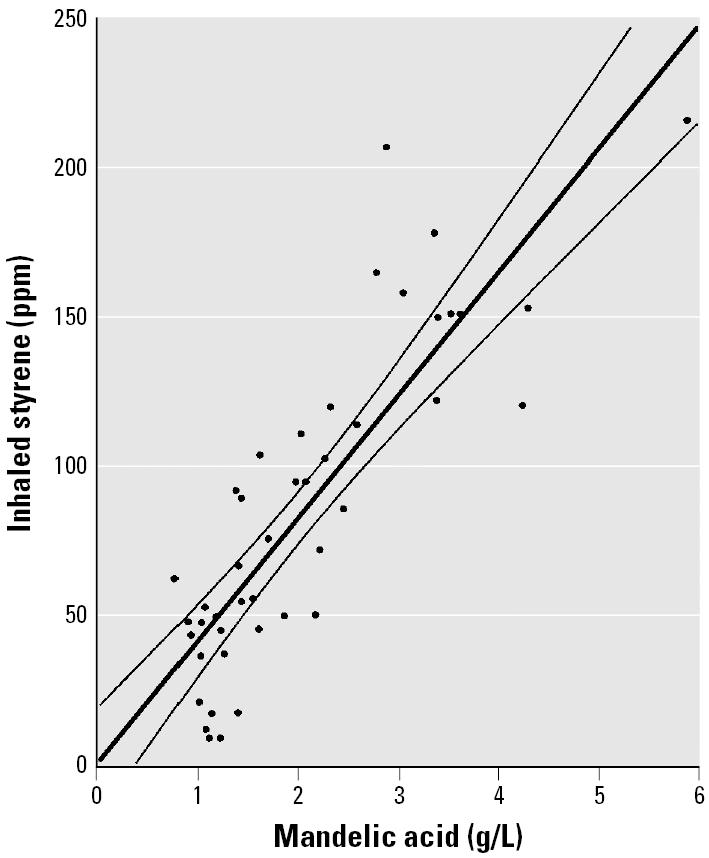
Scatter plot and fitted regression line with 95% CLs for estimation of styrene concentration in inhaled air (TWA ppm) from mandelic acid in urine, normalized by urine volume (g/L). Digitized individual-subject data from one study.

We evaluated the relationship between styrene concentration in inhaled air and styrene concentration in urine from the pooled individual-subject data from five reports (Table 1). These data are presented in Figure 1 along with a linear regression line and 95% confidence limits (CLs). We evaluated the relationship between inhaled-air styrene concentration and mandelic acid in urine (expressed as milligrams per gram creatinine) from the pooled data of three studies (Table 1) and the result is given in Figure 2. Results from the only study (Fields and Horstman 1979) that estimated inhaled-air styrene concentration from mandelic acid (expressed in grams per liter) are shown in Figure 3. Parameters and statistical tests for the three regression lines are given in Table 5. All of the relationships were statistically significant.

### Effects of Styrene on Neurobehavioral Measures

#### Reaction time.

In one case, Mutti et al. (1984) found that urine samples were collected in the morning, just before behavioral testing; in all other cases, urine samples were taken at the end of a work shift. The data for exposures for Mutti et al. (1984) were back adjusted to an end-of-shift value, using a published elimination curve (Engstrom et al. 1976, their Figure 1, group I).

**Figure 4:**
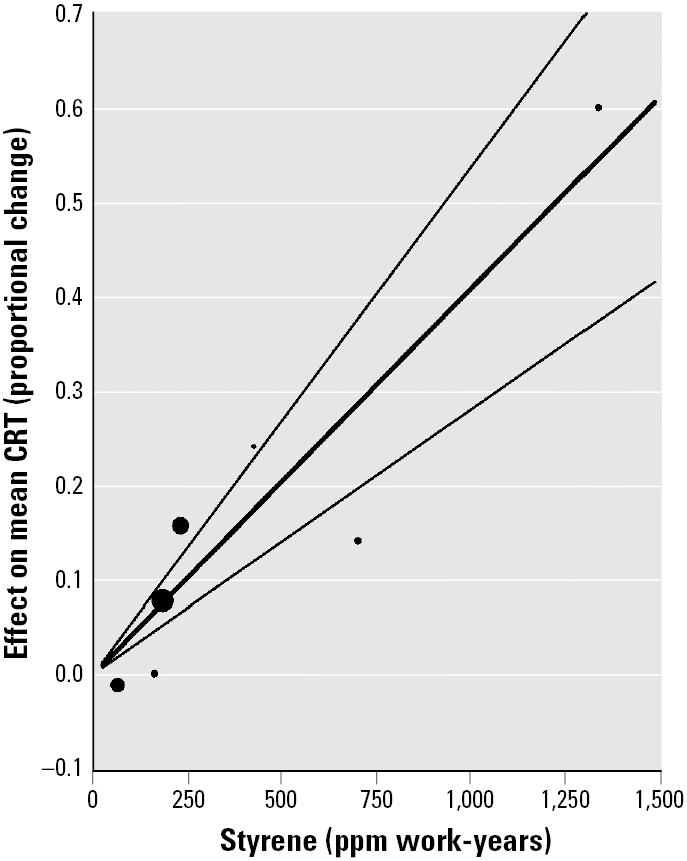
Scatter plot and fitted regression line with 95% CLs for estimation of effect of styrene exposure (ppm work-years) on CRT. Means from five studies were pooled after normalization as in Equation 1. The size of the plotted points indicates the relative number of subjects used in computing each mean.

**Figure 5:**
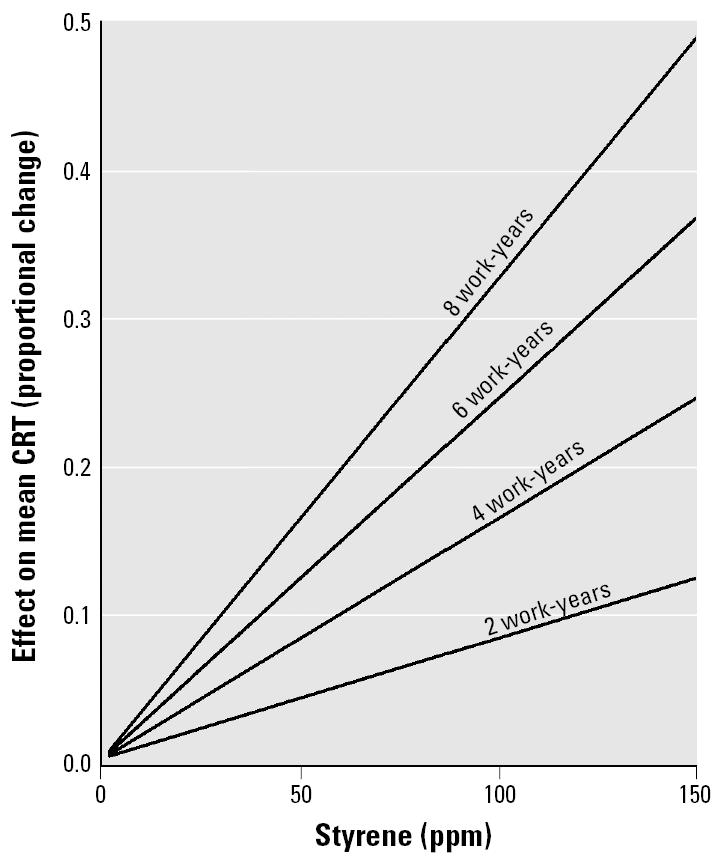
Nomogram for estimating magnitude of effect of styrene (ppm) and work-years of exposure on CRT.

We fitted equation 2 to the pooled mean data for CRT (seven observations) from the studies in Table 2. We observed a statistically significant linear relationship between the mean proportional increase in CRT and cumulative styrene exposure. The intercept term was not statistically significant, and the no-intercept fitted equation was statistically significant, accounting for 91% of the variance (Table 6). The mean data along with the fitted equation and 95% CLs are plotted in Figure 4. The size of the plotted points reflects the relative number of subjects used in computing each mean. Because one of the means in the CRT data was collected at considerably higher exposure (1,336 ppm work-years) and therefore had a much greater effect magnitude (Figure 4), concern arose that the fitted line may have been heavily influenced by this point. We did an exploratory analysis without the extreme point, and the results are given in Table 6 (labeled “CRT, exploratory”). Figure 5 gives the effect magnitude as a function of styrene parts per million for 2, 4, 6, and 8 work-years of exposure (calculated by setting the work-years of exposure to either 2, 4, 6 or 8, and solving for the effect of parts per million with the fitted regression equation).

The relationship between SRT and styrene exposure was not statistically significant in the pooled mean data from three studies (Table 6).

#### Color confusion index.

**Figure 6:**
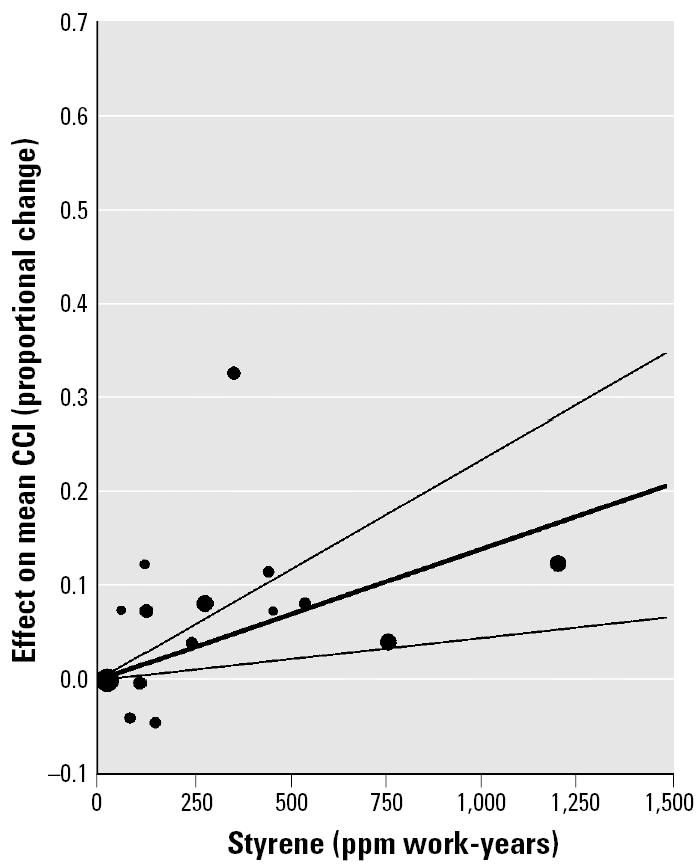
Scatter plot and fitted regression line with 95% CLs for estimation of effect of styrene exposure (ppm work-years) on CCI. Means were computed from digitized individual-subject data, which had been normalized by Equation 1. Means were then pooled from six studies. The size of the plotted points indicates the relative number of subjects in the computation of each mean.

**Figure 7:**
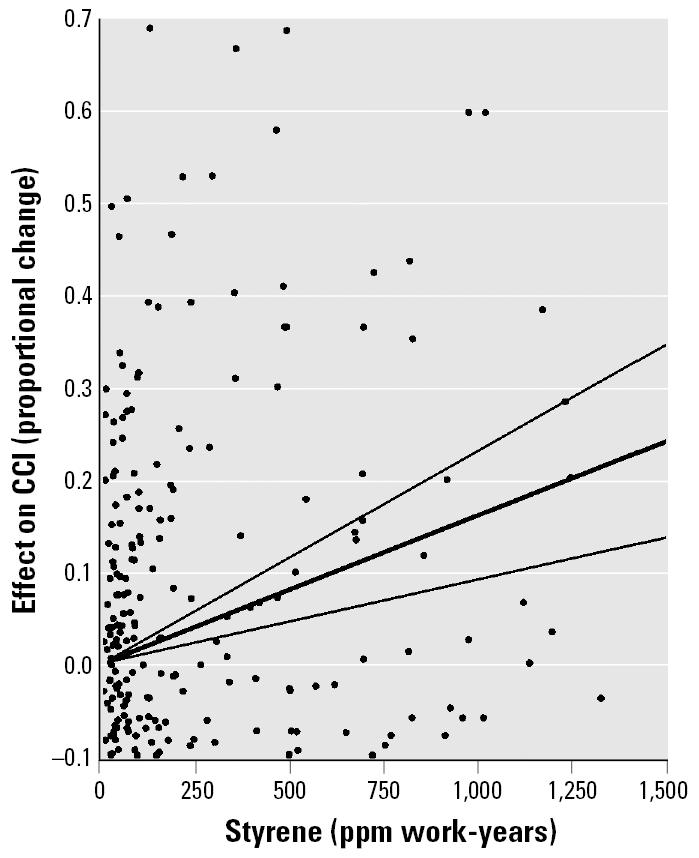
Scatter plot and fitted regression line with 95% CLs for estimation of effect of styrene exposure (ppm work-years) on CCI. Digitized individual-subject data were pooled from six studies.

We fitted a regression equation to mean data (as computed from the individual-subject data) to keep the CCI results comparable with those of reaction time. The intercept term was not statistically significant, and the no-intercept form of the equation was statistically significant and accounted for 35% of the variance (Table 6). Figure 6 is a plot of the mean data along with the fitted line and its 95% CLs. The size of the plotted points reflects the relative number of subjects used in computing each mean. Equation 2 was also fitted to the pooled individual-subject data (329 observations) from the studies in Table 3. The intercept term was not statistically significant and the no-intercept form was statistically significant with the β_2_ term similar to that for the equation fitted to the means (Table 6). Figure 7 is a plot of the individual-subject data and the fitted line with 95% CLs. The confidence limit for individual-subject data is somewhat narrower than for the means (Figure 6). The scale of Figure 7 was kept the same as Figure 6 to facilitate comparisons, even though some of the points were off scale.

**Figure 8:**
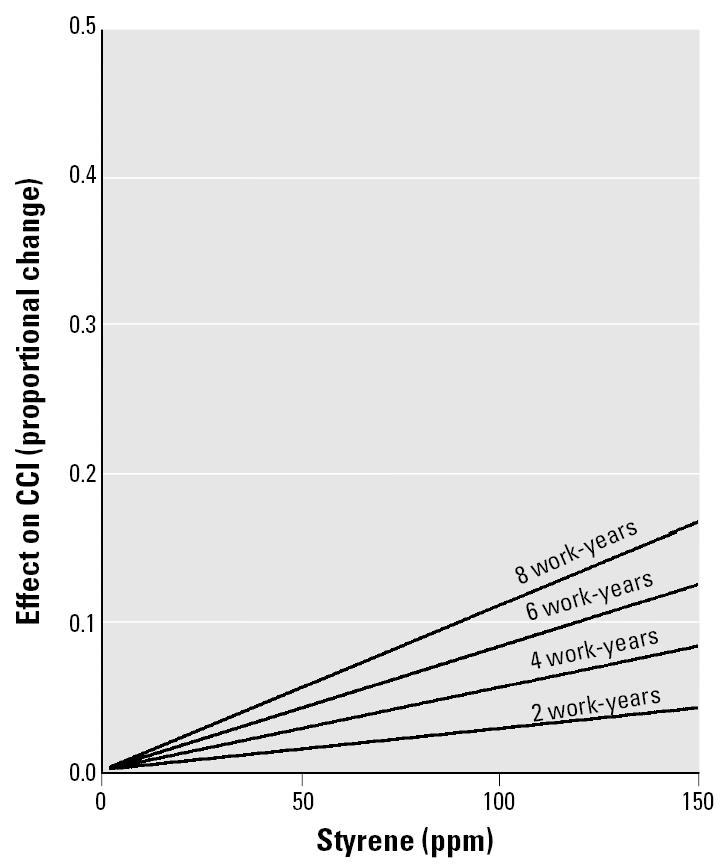
Nomogram for estimating magnitude of effect of styrene (ppm) and work-years of exposure on CCI.

Figure 8 gives the magnitude of effect plotted as a function of styrene ppm for 2, 4, 6, and 8 work-years of exposure (calculated by setting the work-years of exposure to either 2, 4, 6, or 8 and solving for the effect of parts per million with the fitted regression equation). The scales were kept the same as for Figure 5 to facilitate comparison with CRT results.

## Discussion

### Estimates of Exposure

Inhaled-air styrene concentration was linearly related to styrene concentration or its metabolites in urine (Figures 1–3). Inspection of these figures reveals that, although the equations fit well, a number of individual-subject data lay outside the confidence limits. One potential source of such errors is the measurement of inhaled-air styrene, which was usually made with dosimeters placed “near” the subject’s personal exposure space and might not have measured actual exposure. Another potential source of variance involves differences in physical activity across subjects, which would have affected the amount of styrene inhaled. Despite the observed variability, the overall trend and the statistical significance of the fitted lines in Figures 1–3 reveal that all three biomarkers of styrene exposure provide reasonable indicators of recent exposure.

### Behavioral Effects

#### Choice reaction time.

Cumulative styrene was associated with increased CRT in a dose-related manner. Inspection of Figure 4 reveals that one point lies at a higher exposure value with respect to the others. That point is the mean for one of four groups from the same study, each exposed to a different amount of styrene. These four means are represented as the smallest four points in Figure 4. They form a series that is consistent with the fitted dose effect function. Each of the points is the mean of 18–28 subjects, for a total of 100 subjects. Despite the fact that one point is outstanding in Figure 4, the fact that it came from the only study with multiple exposure levels makes the resulting fitted equation plausible. Furthermore, an exploratory analysis, with the extreme point removed from the data set, yielded only a slightly lower slope and a poorer fit. The fitted line from the exploratory analysis was well within the confidence limits of the line fitted to the whole data set. More data at the upper end of exposure would improve the confidence limits.

No significant effects were observed on SRT, perhaps because the largest exposure for the SRT data was only about 250 ppm work-years. Thus, there may not have been sufficient exposure to produce a reliably detectable effect.

Under the assumption that the metric of exposure can be separated into discrete concentration (parts per million) and duration (work-years) components, the lines in Figure 5 may be used to estimate the magnitude of various exposure histories on CRT. For example, 8 work-years at 150 ppm is estimated to produce an increase in CRT of almost 50%. For 20 ppm, a contemporary limit for occupational styrene exposure [American Conference of Governmental Industrial Hygienists (ACGIH) 2000] for 8 work-years is estimated to produce a 6.5% increase in CRT. By use of the fitted dose–effect equation the increase in CRT can be estimated for any combination of concentration and duration of exposure.

#### Importance of rapid reaction times.

The importance of reaction times has been discussed in a number of ergonomic settings, among which perhaps the most quantitative is automobile driving. For drivers in the United States, it has been estimated that reducing the reaction time by 100 msec would reduce accident-related property damage costs alone by $655,000,000 annually (1994 US$) and prevent 58,000–70,000 injuries per year (Blincoe 1994; Kahane and Hertz 1998). For unexpected events, a decrease in reaction time of 100 msec is about 7% of the normal reaction time, and for expected events 100 msec is about 14% (Green 2000). Thus, changing reaction time by 7–14% has important economic and personal implications. Styrene exposure to permissible levels produced the magnitude of change upon which the above economic estimates were based. In addition, consideration should be given to the possibility that people sometimes have additionally increased reaction times due to work-related fatigue and consumption of ethanol and drugs.

Given the above information it would be possible to calculate the benefit of any proposed changes in styrene exposure limits if data were available on *a*) the number of workers exposed to styrene, *b*) the distribution of exposure concentrations and durations, and *c*) the duration of the effect after cessation of work-place exposures. It would then be possible to compare the cost of regulation to the benefit of such regulation on a continuous dose-related scale. Presumably, the above data could be found, except for estimates of the permanence of the increased CRT after exposure cessation.

#### Color vision.

Long-term exposure to styrene was associated with increased errors in performing a color discrimination/arrangement task in the pooled data from six studies of occupationally exposed workers. The mean effect size for CCI was estimated with closer confidence limits for Figure 7 than for Figure 6 because of the use of individual-subject data, but the regression lines for the two procedures were similar. The individual-subject observations scattered widely about the estimated line, lowering the variance accounted for. For an exposure of 8 work-years to 150 ppm, the estimated increase in CCI score was approximately 17% (Figure 8). This is lower than for CRT, which was estimated at nearly 50%. For 20 ppm, a typical limit (ACGIH 2000) for 8 work-years, there was an estimated 2.23% increase in CCI.

Color vision deficiencies associated with exposure to styrene and other solvents have been associated primarily with difficulty in discriminating among colors at the “blue” end of the spectrum. This is commonly referred to as a blue/yellow deficit. This type of color vision deficit could be associated with reduced function in the short-wavelength-sensitive (blue) cones or their associated ganglion cells (Greenstein et al. 1990; Hood et al. 1984; Pachec-Cutillas et al. 1999). Why, or if, these cones are actually more susceptible is not well understood.

The measures of CCI show a relatively large variance among individual subjects, as was observed in group mean data by Paramei et al. (2004). Factors that could contribute to this variance include use of incorrect chromaticity values derived from the saturated Farnsworth-Munsell D-15 test, rather than unsaturated D-15d test (Geller 2001; Geller and Hudnell 1997) and differences between spectrum color profiles of light sources labeled as “daylight” (Wyszecki and Stiles 1982). Also important are the effects of luminance and practice that were not necessarily constant across studies.

#### Importance of color-vision deficits.

The importance of impaired color perception is difficult to specify quantitatively. The broad scope of tasks deleteriously affected by color confusion should, by itself, give weight to the importance of deficits. Color information is important to persons who are driving; making distance judgments; reading colored text on video monitors, medicine bottles, food cans, and the like; scanning for objects in a complex visual scene; or working with color-coded electrical circuitry (Klein et al. 1999; McClure et al. 2000; Owsley et al. 2001; Rubin et al. 1994, 1997, 2001). Congenital color deficiencies are related to poorer school performance, slowed CRT at traffic lights, difficulty with information processing from color video monitors, and increased difficulty with color-coded tasks (Cole and MacDonald 1988; Margrain et al. 1996; O’Brian et al. 2002; Steward and Cole 1989). Although all of these tasks are important, there is no obvious way of relating these reported deficits to the magnitude of effects reported for styrene. For such a relationship to be established, experimental evidence is needed to relate CCI values to task performance.

It is possible to compare the effect of styrene exposure with the effect of aging on CCI. The CCI of men increases with age at the rate of about 10% of baseline every 13 years of age (Iregren et al. 2002, their Figure 1 and Table 6). Thus, the deficit in color perception caused by exposures to styrene of 115 ppm for 8 work-years or 156 ppm for 6 work-years is roughly equivalent to 13 years of additional age in visual dysfunction. Eight work-years at 20 ppm (the ACGIH limit) would produce a 2.23% deficit, which is roughly equivalent to 1.7 additional years of age.

### Reversibility of Effects

It is not clear whether the effects of long-term styrene exposure are reversible. All of the data analyzed in the present work were collected at least 15 hr after the last exposure and therefore are probably not due to the concurrent presence of styrene or its metabolites in the blood. Some experimenters gave behavioral tests to subjects both before and after a day’s exposure and found no statistically significant differences for CRT (Jegaden et al. 1993; Triebig et al. 1989) or for CCI (Triebig et al. 2001). This implies that the acute body burden of styrene and its metabolites was not responsible for the effects and that longer-term processes were acting.

A few of the experimenters tested subjects both before and long times after exposure, to characterize the reversibility of effects. For CCI, Triebig et al. (2001) reported that effects were reversible after 4 weeks of vacation. This study had relatively low exposures (even though the effect was large). Mergler et al. (1996) reported that reduction of exposure due to workplace improvements also reduced the CCI effect (and other behavioral effects) after a period of 2 years. These data are scant and spotty but suggest the possibility that at least some recovery may occur from the effects of long-term exposure to styrene.

### Possibility of Estimation Errors

It is possible that the concentration of styrene in the past was greater, by an unknown amount, than indicated by contemporary measures because workplace improvements may have been made (Gong et al. 2002). If this were the case, then the noted effects would have been due, in part, to higher styrene exposures in the past and not to the possibly lower concurrent exposures. Although this would have the effect of overestimating the magnitude of effect for any indicated concentration of exposure, it would not have affected the statistical significance of the finding that styrene produces the indicated effect.

On the other hand, all of the available data in this analysis were from studies of occupationally exposed workers. The risks of chronic styrene exposure to the general population may have been underestimated to the extent that healthy workers are not representative of the general population. It is possible that persons who are more susceptible to effects of styrene exposure do not remain in positions where such exposure occurs—the so-called “healthy-worker effect.” It is also possible that nonworking persons such as the young or elderly might be more susceptible to effects of styrene exposure than are healthy workers.

## Summary and Conclusions

Workplace styrene exposure can increase CRT and CCI. The magnitudes of the effects are statistically significant linear functions of parts per million work-years. The magnitude of each effect is a continuous function and reaches socially important values at realistic exposure concentrations.

Increased CRTs are associated with impairment of tasks performance, such as driving, that can be monetized for benefit–cost analysis. Increased color vision deficiencies are associated with difficulty in the performance of many everyday tasks. The cost of these increases is difficult to estimate. It appears that the effects on CRT and CCI persist for some time after exposure ends, but this conclusion is based on limited data.

The effects of styrene on CRT and CCI may have been overestimated by an unknown amount in this meta-analysis because of *a*) underestimates of past exposure and *b*) bias from experimenter knowledge of subject exposure status while testing. On the other hand, the potential for effects of styrene exposure in the general population may have been underestimated because of the healthy-worker phenomenon or because of the lack of susceptible persons in the workplace, such as the young or elderly.

## Figures and Tables

**Table 1:** Studies of styrene urinary measures as a function of styrene exposure.

Reference	Urinary measure	No.
Campagna et al. 1995	Mandelic acid (mg/g creatinine)	58
De Rosa et al. 1993	Mandelic acid (mg/g creatinine)	22
Fields and Horstman 1979	Mandelic acid (g/L urine)	44
Ghittori et al. 1987	Styrene	88
Gobba et al. 1993	Styrene	193
Imbriani et al. 1986	Styrene	92
Imbriani et al. 1990	Styrene	25
Pezzagno et al. 1985	Styrene	93
Severi et al. 1994	Mandelic acid (mg/g creatinine)	66

**Table 2:** Studies of styrene effects on CRT.

Reference	Exposure measure	CRT measure	Control?	Blind?	No.	Comment
Jegaden et al. 1993	MA in urine, end of shift (reference text)	Preexposure (Table 2, “morning”)	Yes	?	60	Means only
Mutti et al. 1984	MA in urine, at behavior test time (reference text)	Preexposure (Table 2)	Yes	?	100	Four independent means; exposure back calculated; see reference text
Triebig et al. 1989	MA in urine, end of shift (Table 3 and reference text)	Preexposure, (Table 4, CRT, group A)	Yes	?	56	Means only
Tsai and Chen 1996	Inhaled air concentration (Table 1)	Preexposure, (Table 4, mean of tests 2, 5, 6, and 8)	Yes	Yes	86	Means only

Abbreviations: ?, information not given in the referenced text; MA, mandelic acid. Figures, tables, and text listed in the table refer to source of data in reference cited.

**Table 3:** Studies of styrene effects on SRT.

Reference	Exposure measure	SRT measure	Control?	Blind?	No.	Comment
Gamberale et al. 1976	Inhaled air concentration, (Tables 1 and 2)	Preexposure, (Table 3, morning values)	Yes	?	142	Four independent sites, one data point each; means only
Jegaden et al. 1993	MA in urine, end of shift (see reference text)	Preexposure (Table 1, “morning”)	Yes	?	60	Means only
Triebig et al. 1989	MA in urine, end of shift (Table 3 and reference text)	Preexposure, (Table 4, SRT, group A)	Yes	?	56	Means only

Abbreviations: ?, information not given in the referenced text; MA, mandelic acid. Figures, tables, and text listed in the table refer to source of data in reference cited.

**Table 4:** Studies of styrene effects on CCI.

Reference	Exposure measure	CCI measure	Control?	Blind?	No.	Comment
Campagna et al. 1996	Inhaled air, 2nd shift (Table 1)	Preexposure (Figure 1)	No	?	128	Raw data and means
Eguchi et al. 1995	MA in urine, end of shift (Table 1)	Preexposure (Table 1 and Figure 3)	Yes	?	62	Raw data and means
Gobba 1991; Gobba and Cavalleri 2000	Inhaled air, during shift [Table 1 and reference text (Gobba 1991)]	Preexposure [Figure 1 (Gobba and Cavalleri 2000)]	Yes	Yes	51	Raw data and means in Gobba and Cavalleri 2000; details in Gobba 1991
Gong et al. 2002	MA in urine, end of shift (Figure 2, Table 1)	Preexposure (Figure 3)	Yes	Yes	55	Raw data and means
Kishi et al. 2001	MA in urine, end of shift (Table 1)	Preexposure (Table 2 and Figure 2)	Yes	?	87	Raw data and means

Abbreviations: ?, information not given in the referenced text; MA, mandelic acid. Figures, tables, and text listed in the table refer to source of data in reference cited.

**Table 5:** Statistics for exposure-estimation equations.

Equation	*p*-Value	β_1_	β_2_	*R*^2^
Styrene concentration in urine	< 0.0001	−1.749	0.6190	0.726
Mandelic acid in urine (mg/g creatinine)	< 0.0001	0.289	0.0793	0.838
Mandelic acid in urine (g/L urine)	< 0.0001	30.500	41.1000	0.851

The *p*-value is from the *F*-test for overall fit, the βs are the equation parameters, and *R*^2^ is the corrected value of squared correlation.

**Table 6:** Statistics for dose–effect equations.

Equation	*p*-Value	β_1_	β_2_	*R*^2^
CRT	0.0002	−0.0209, *p* = 0.672	0.000408, *p* = 0.0002	0.909
CRT, exploratory	0.0139	—	0.000327, *p* = 0.0139	0.733
SRT	0.5680	—	—	—
CCI, means	0.0062	0.0344, *p* = 0.326	0.000139, *p* = 0.0062	0.348
CCI, raw	< 0.0001	0.0040, *p* = 0.821	0.000184, *p* < 0.0001	0.072

—, parameter not statistically significant. The *p*-value is the *F*-test result for the final form of the fitted equation. The *p*-value for β_1_ is the *t*-test value for that parameter. If the β_1_ was not significant, the term was not included in the final form. The *p*-value for β_2_ is the *t*-test value for the final form. The *R*^2^ is the value for the final form.
